# Impact of the COVID-19 pandemic on mental health care and people with mental health conditions in Ethiopia: the MASC mixed-methods study

**DOI:** 10.1186/s13033-023-00612-8

**Published:** 2023-12-06

**Authors:** Awoke Mihretu, Wubalem Fekadu, Azeb Asaminew Alemu, Beakal Amare, Dereje Assefa, Eleni Misganaw, Abebaw Ayele, Ousman Esleman, Zewdu Assefa, Atalay Alem, Graham Thornicroft, Charlotte Hanlon

**Affiliations:** 1 Department of Psychiatry, WHO Collaborating Centre for Mental Health Research and Capacity-Building, School of Medicine, College of Health Sciences, Addis Ababa University, Addis Ababa, Ethiopia; 2 Mental Health Case Team, Disease Prevention and Control Directorate, Ministry of Health, Addis Ababa, Ethiopia; 3 Mental Health Service Users Association, Addis Ababa, Ethiopia; 4 Policy, Plan and Monitoring & Evaluation Directorate, Ministry of Health, Addis Ababa, Ethiopia; 5 Center for Public Health Emergency Management, Ethiopian Public Health Institute, Addis Ababa, Ethiopia; 6 Centre for Global Mental Health, Department of Health Services and Population Research, Institute of Psychiatry, Psychology and Neuroscience, King’s College London, London, UK

**Keywords:** COVID-19, Mental health, Mental health conditions, Stigma, Service utilization, Ethiopia

## Abstract

**Background:**

The COVID-19 pandemic has had far-reaching effects on the mental health of populations around the world, but there has been limited focus on the impact on people with existing mental health conditions in low-income countries. The aim of this study was to examine impact of the pandemic on mental health care and people with mental health conditions in Ethiopia.

**Methods:**

A convergent mixed methods study was conducted. We systematically mapped information from publicly available reports on impacts of the pandemic on mental health care. Monthly service utilisation data were obtained from Amanuel Mental Specialised Hospital, the main psychiatric hospital, and analysed using segmented Poisson regression (2019 vs. 2020). In-depth interviews were conducted with 16 purposively selected key informants. Framework analysis was used for qualitative data. Findings from each data source were integrated.

**Results:**

In the early stages of the pandemic, participants indicated a minimal response towards the mental health aspects of COVID-19. Mental health-related stigma and discrimination was evident. Scarce mental health service settings were diverted to become COVID-19 treatment centres. Mental health care became narrowly biomedical with poorer quality of care due to infrequent follow-up. Households of people with pre-existing mental health conditions in the community reported worsening poverty and decreased access to care due to restricted movement, decreased availability and fear. Lack of reliable medication supplies increased relapse and the chance of becoming chained at home, abandoned or homeless. Caregiver burden was exacerbated. Within mental health facilities, prisons and residential units, infection control procedures did not adequately safeguard those with mental health conditions. Meanwhile, the needs of people with mental health conditions in COVID-19 quarantine and treatment facilities were systematically neglected. Only late in the day were integrated services developed to address both physical and mental health needs.

**Conclusions:**

The COVID-19 pandemic had substantial negative impacts on the lives of people with mental health conditions in Ethiopia. Future emergency response should prioritise the human rights, health, social and economic needs of people with mental health conditions. Integration of mental and physical health care would both expand access to care and increase resilience of the mental health system.

**Supplementary Information:**

The online version contains supplementary material available at 10.1186/s13033-023-00612-8.

## Background

People with mental health conditions may have been disproportionately adversely affected by the COVID-19 pandemic due to longstanding marginalization and stigma alongside pre-existing health and economic vulnerabilities [1, 2]. The precarious state of mental health systems and social care in many low- and middle-income countries is anticipated to have heightened these negative impacts, but there have been few reports examining the impacts of the pandemic on mental health care and people with pre-existing mental health conditions in low-resource settings [3, 4].

Prior to the COVID-19 pandemic, the prevalence estimates for all major mental health conditions in Ethiopia were comparable to global estimates. This included severe mental health conditions (e.g., schizophrenia and bipolar disorder [5–7], depression [8], alcohol and substance use problems [9] and child mental health conditions [10]. The impact of untreated mental health conditions in Ethiopia pre-pandemic was substantial [11], including disability [12], premature mortality [13, 14], chaining or restraint [15, 16], homelessness [17], and impoverishment of affected households [18–20].

Despite the high mental health burden, there was also a high pre-pandemic treatment gap for mental health care in Ethiopia, with an estimated 12-month treatment gap of 60% [11]. Scarcity of mental health professionals in mental health practice, inaccessibility of centralized mental health facilities, high levels of mental health stigma and low levels of community awareness were key contributors to this treatment gap [11]. According to the WHO Mental Health Atlas 2017 report, compared to the WHO recommendation of 0.90 psychiatrists, 8.34 nurses and 7.85 psychosocial workers per 100,000 population, Ethiopia had 0.11 psychiatrists, 0.58 master’s level practitioners/psychiatric nurses, 0.045 clinical psychologists and 0.009 social workers per 100,000 population pre-pandemic^20^. Specialist mental health care is exclusively available in urban settings, while over 80% of Ethiopia’s population lives in rural areas.

Efforts to expand access to mental health care are enshrined in the Ethiopian National Mental Health Strategy with a focus of integrating mental health care into general health and primary health care settings [21]. However, most health centres in rural settings were not delivering mental health care pre-pandemic, even though implementation research studies had shown that integrated mental health care at the district level in Ethiopia can substantially increase treatment coverage [22] and improve clinical, functional, social and economic outcomes of people with mental health and substance use conditions [16, 23–25]. The new Ethiopian Primary Healthcare Clinical Guidelines include horizontally integrated mental health care and offer potential to support scale-up but are not fully implemented [26]. Efforts to expand access to psychosocial care for people with mental health conditions are also underway but have not been taken to scale [27–30].

Ethiopia’s COVID-19 preparedness and response efforts were co-ordinated by activating a national public health emergency operation center as early as January 27, 2020. On March 11, 2020, COVID-19 was declared as a pandemic. Just two days later, the first case of COVID-19 was reported in Ethiopia. Measures to reduce the spread of infection were initiated, which included school closures, suspension of international flights and partial restriction of public gatherings. These measures were followed by a state of emergency declaration on April 8, 2020, which extended the restrictions up to August 2020. Additional restrictions were introduced on public gatherings, including at religious centers [31]. Although several activities targeting mental health and psychosocial support were implemented [32], the extent to which these measures mitigated impacts of the pandemic on people with existing mental health conditions (MHCs) and mental health care in Ethiopia was not known. There have been several studies focused on the mental health impacts of COVID-19 on various segments of the general population in Ethiopia [33–36], but we managed to find only one study which explored its effects on individuals with pre-existing mental health conditions. That study was restricted to a single rural setting and reported lower levels of health and economic wellbeing, increased stress, greater susceptibility to COVID-19, and heightened stigma and exclusion among people with severe mental illness. Additionally, the study found that mental health service utilization was low among this population [37].

The Mental health care: Adverse Sequalae of COVID-19 (MASC) study is a multi-country study carried out in Chile, Ethiopia, Georgia, Nigeria, South Africa, Sri Lanka and Ukraine. The MASC study will produce comparable evidence on the impact of the COVID-19 pandemic on people with MHCs across diverse LMICs. Evidence from MASC will be used to advocate for an improved mental health response to COVID-19, both at the national (e.g. within Ethiopia) and international levels. The aim of MASC was to identify the impacts of COVID-19 on mental health care and people with mental health conditions, the innovative responses employed to maintain mental health care and the lessons learned. In this paper, we present MASC study findings from Ethiopia.

## Methods

### Study design

We conducted a convergent mixed-methods study. We convened a national expert group (including people with lived experience of mental health conditions, policymakers, practitioners, non-governmental organisations, religious leaders and human right experts) to oversee the process, to map publicly available data sources using a customized narrative tool and to contribute to the synthesis and interpretation of the findings. Alongside this, we conducted a qualitative study comprising semi-structured interviews with key informants about the perceptions and experiences about mental health and the impacts of COVID-19 in Ethiopia. Health management information system data on mental health service utilization were also obtained. All data sources were triangulated and analysed in relation to a pre-specified framework.

### Qualitative study sample and sampling

Key informants were purposively selected from the Federal Ministry of Health of Ethiopia, the emergency response team of the National Public Health Emergency Operation Centre at the Ethiopia Public Health Institute, the World Health Organization (WHO) country office, leads for mental health services in Addis Ababa and regional centres (public and private), mental health service user and caregiver representatives, representatives of health worker associations, advocates and members of civil society organizations. Selection was augmented with a snowballing approach through members of the technical working group for Mental Health and Psychosocial Support to identify non-governmental organizations involved in care of people with mental health conditions.

### Data sources and data collection instruments

We employed three types of data sources: a narrative tool, semi-structured interviews and health management information system data.

#### Narrative tool

We developed a narrative tool to identify all information available in relation to: [1] provision of, and access to, services for people with mental health conditions (including both physical and mental health care); [2] quality of services (including staffing ratios, hours of provision, lowered frequency or quality of safety or quality monitoring); [3] delivery of health care services and systems (including changes to mental health law, arrangements for patients or residents to agree to ‘not for resuscitation’ orders, reductions in mental health budgets to subsidize the COVID-19 response; or less access to personal protective equipment for mental than physical health care staff); [4] exemplars of good practice on how to mitigate adverse impacts on mental health services during the COVID-19 pandemic. Respondents were asked to provide a narrative account of their experiences, backed up by evidence sources where available. See Supplementary File 1.

#### Topic guide for semi-structured interviews

Our semi-structured topic guide covered perspectives on the impact of COVID-19 on mental health care at all levels in the health system and the multi-dimensional effects on people with mental health conditions and their caregivers. The domains were the same as those described for the narrative tool.

#### Health management information system data

Monthly routine mental health service utilization data were obtained for 2019 and 2020 from Ministry of Health of Ethiopia, and from the largest hospital providing mental health care in Ethiopia (Amanuel Mental Specialized Hospital). The ethical approval obtained from Addis Ababa University covered use of this dataset. Data cleaning is performed by the hospitals. For the MASC analyses we used raw, untransformed data.

### Data collection procedures

Semi-structured interviews were conducted virtually (n = 2) or face-to-face (n = 16) in English or Amharic by PhD level interviewers with experience in qualitative data collection. Interviewees (n = 17) were initially contacted by phone or email, and further clarification or interview scheduling was conducted though phone conversation. Written, informed consent was obtained. Interviews were audio-recorded, with permission. Amharic transcripts were translated into English.

### Data analysis

For the qualitative data, a framework approach to analysis was used [38]. See supplementary file 1 for the framework, which was based on the domains of the narrative tool described above. We repeatedly read the transcribed interviews to develop familiarity with the dataset. We then developed a data coding system and linked codes to domains of our framework. Framework analysis enables a transparent data analysis process, progressing through a series of interconnected stages that allow the researcher to analyse the data in an iterative way. The monthly service utilization data were analysed using segmented poisson regression comparing 2019 (pre-pandemic) to 2020. Findings from the different data sources were then integrated in relation to the narrative tool domains.

### Ethical considerations

#### Ethical approval

was obtained from the Institutional Review Board of the College of Health Sciences, Addis Ababa University (Ref. 087/20/CDT). In addition to usual ethical considerations, we also adhered to the standard national guidelines for COVID-19 precautions, including wearing face masks, physical distancing and taking hand hygiene measures during data collection.

## Findings

Interviews were conducted with 18 key informants. See Table 1 for the categories of respondent.

**Table 1:** Characteristics of respondents

Category	Participant number and gender
Service users	1 male and 3 females
Representatives from national bodies involved in mental health response to COVID-19	2 males
Mental health experts (Masters level Mental Health professionals, psychiatrists, leaders of mental health professional associations).	8 Males
Religious leader	1 Male
Human rights advocate	1 Male
Civic society representative (a representative from either civic society or non-governmental organizations)	2 Males

Our findings are structured in relation to: (1) impacts of COVID-19 on people with MHCs in the community, prisons and residential settings, (2) mental health-related stigma and social exclusion, (3) mental health in COVID-19 quarantine, isolation and treatment settings, (4) impact of COVID-19 on the physical health of people with MHCs, and (5) changes in access and quality to mental health care.

### Impacts on people with MHCs in the community, prisons and residential settings

Key informants involved in service provision and mental health service users reported that COVID-19 seriously affected the recovery process of people with MHCs. Among other factors, the disruption of medication supplies, economic burden and loss of social networks and support impacted the mental health recovery of individuals with MHCs. Respondents noted that many people with MHCs had disengaged from the daily routines that had previously served as important coping mechanisms. Inability to access their support networks and the absence of emotional supports were also reported to have impacted their mental health.

When we [service users] meet physically there is gesture, body language, hug, and human touch. We lost all of these. For a service user, these and our support networks, in general, are our copings but this has been impacted by COVID-19. This has also an impact on our recovery process [Service user; IDI01].

COVID-19 prevention and control measures were also reported to have impacted the lives of individuals with substance use problems. There was a perception that shops selling alcohol were closed, so individuals with alcohol use disorders suffered from alcohol withdrawal, especially during the state of emergency. Those who were in the process of recovery from substance use problems were also considered to be vulnerable to relapse due to lack of access to their social networks and rehabilitation services.

Participants disclosed that people with existing MHCs or other people in need in the community were not adequately supported to maintain their mental health. One reported reason was that mental health had not been integrated into community platforms in Ethiopia. As mental health care and support was only available in centralized facilities, the infrastructure to reach people with MHCs in the community during COVID-19 did not exist.

MHPSS [mental health and psychosocial support] activities were not reaching into the community except limited public communications using radio [Mental health expert; IDI02].

Respondents reported increased household burden and economic strain due to the COVID-19 pandemic, in some cases leading to abandonment and homelessness of people with MHCs. Caregivers shared experiences of the challenges of conveying a person with a MHC to hospital. The movement restrictions, increased transportation costs and costs of commodities contributed to the economic burden.

Transportation cut down in half; means one passenger in two seats. For example, if a patient comes from far and used to pay 100 Birr [about 2USD] now he/she pays 200 Birr and for round trip means 400 Birr, if it was 50 Birr now its 200 Birr for round trip [Service user; IDI08].

Mental health professionals also recognized the burden on caregivers. They explained that since hospital admissions had been suspended, caregivers were required to take full responsibility for caring for people with MHCs when they were acutely unwell. During enforcement of the ‘stay-at-home’ regulation, caregivers were alone in providing full-time care and monitoring of their family members with a mental health condition.

There were also reported additional economic strains on people with MHCs due to COVID-19. As many individuals with MHCs were engaged in irregular income-generating activities or were dependent on their families, they were disproportionately affected by restrictions on movement and trading. Participants highlighted that, since there is a long-standing public perception that people with MHCs are unable to work or have reduced capacity, when employers reduced the number of employees during COVID-19, people with MHCs were the first victims.

My mother [spouse of a man with a severe mental health condition] is a trader. She is the one who buys things for our family. When the illness comes, she could not go to the market. You can imagine. It was very difficult for our family. He cannot help her [Service user; IDI03].

Although there were several general community initiatives to provide support for economically vulnerable sections of society, there were no data on whether people with MHCs were included. Respondents expressed the need to advocate for people with MHC and their families to be prioritized for community social, food and financial support initiatives on an ongoing basis during the pandemic.

Social care institutions providing services for the homeless and destitute reported increased numbers of people with MHCs needing their care during the pandemic. As COVID-19 infection control measures also applied to religious institutions, holy water sites in Addis Ababa were closed for some time during the early months of the pandemic. As a result, some people with MHCs who had no access to family and who would otherwise have resided within holy water sites became homeless.

The informal sector, such as holy water, accommodates the largest proportion of people with mental illness and these mental health care options have been closed due to COVID-19, of course, they are recently opening. Thus, patients could be abandoned and go to street life [Mental health expert; IDI04].

Non-governmental organisations providing care for those who were destitute also struggled due to reduced availability of volunteers. To reduce the risk of coronavirus infection, such organisations were forced to accommodate the caregivers for extended periods (about 3 months) which also incurred additional expenses. Due to COVID-19, these organisations had to construct or rent more rooms so that they could physically distance their beneficiaries. At the same time that they faced increasing costs, social care organisations reported reduced donations during COVID-19.

Since there was an economic impact of COVID-19 among everyone and local donors have also been economically challenged, our organisation has got financial constraints for operational costs, employees’ payments and buying medications. As you know psychiatric medications are expensive and the number of our beneficiaries has also increased to 1500 [Civic society representative; IDI05].

Some COVID-19 protocols of residential institutions impinged upon the rights of people with MHCs. The following quote illustrates the lack of parity between beneficiaries with mental health rather than physical health conditions who were residing in a social care institution.

Yes, we did operational changes. At some point, we had suspended social visits to our organisation. But for the survival of the organisation, to sustain donations, we then resume it. But visitors were not allowed to visit psychiatric rooms and see individuals with MHCs because they did not wear face masks [Civic society representative; IDI06].

Disruption of mental health care and other human rights violations were also reported among people with MHCs during COVID-19 in prisons.

During COVID-19, we [EHRC office] did an assessment to monitor how the Ministry of Health COVID-19 prevention and control measures are implemented without affecting the human rights of prisoners. We found that prisoners with mental health care needs were not getting treatment and not taking their regular medications. This is due to movement restriction and the police officers fear of the infection when travelling individuals with mental health need to health care facilities. Meals were also not sustainably delivered on time. Women were also not sustainably getting sanitary pads [Human rights advocate; IDI07].

### Mental health-related stigma and social exclusion related to COVID-19

Long-standing mental health stigma and social exclusion served to exacerbate the adverse impacts of COVID-19 on the lives of some people with MHCs. The high levels of stigma towards people suspected of having COVID-19 or those who had recovered from COVID-19 intersected with community attitudes that people with MHCs were unpredictable and unreliable. Consequently, in some communities, people with MHCs were perceived as an infection threat. Community members kept their distance from households of a person with a MHC or harassed individuals with MHCs in relation to their possible non-adherence to COVID-19 prevention and control measures. Sometimes people with MHC were restrained due to concerns over vulnerability to infection.

Because my husband, who has a severe mental health condition, fails to wear the face mask, once he had a quarrel with a police officer because he refuses to wear the mask. He wears the mask by now, but he may not do it properly. People were distancing him [her husband], thinking as he may not protect himself [Service user; IDI08].

Other respondents emphasized that human rights abuses of people with MHCs had pre-dated the pandemic.

Before COVID-19, people with mental health conditions are not respected and fulfilled. Thus, if you ask me if COVID-19 exacerbated this, I don’t think. The problem was there, before COVID-19 and could be ongoing even after COVID-19 [Mental health expert; IDI09].

On the other hand, respondents observed that COVID-19 had put mental health on both the national and international agenda. This had created opportunities for open discussion about mental health which were not present before the pandemic.

There were many interviews over the media about mental health. Thus, now talking about anxiety and self-care is not a shame. Normalizing the discussion about mental health is one good thing, but it was not sustained [Service user; IDI01].

Religious leaders were among those who recognized the importance of addressing mental health stigma.

Being human is esteemed by God. You are not allowed to abandon someone for his/her mental health condition because he/she is a human being. No one is immune to illness. No one is absolutely healthy on earth. We all were sick or potentially sick. I personally had experience insanity. I went to the street for three years and was naked when I was a child. Later, God had said that it is enough and restored me to my current position. So, everybody shouldn’t ignore or stigmatize people with MHCs. The one who looks healthy could be insane and the insane will also be healthy. To stigmatize is to lack God’s grace. It is better if people live with faith and love [Religious leader; IDI10].

### Mental health in COVID-19 quarantine, isolation and treatment settings

Distressing experiences were reported for many who were admitted to COVID-19 quarantine (those waiting for test results after travel) or isolation centres (those waiting for test results after showing symptoms), but with particular impacts on vulnerable groups (e.g. domestic workers and returnees from the Middle East) and on those with existing MHCs.

One individual had thrown away himself from a building and fell down on a water rotor. His leg and his teeth were broken. There was also another one who fell down from a building. The first one was a returnee from Middle-East. When he was later evaluated by the clinicians, he had been experiencing major depression symptoms. The second one had also been taking psychiatric medications before deportation. There were more suicide attempt incidents in quarantine centres and I know about four incidents [National COVID-19 mental health response; IDI11].

Mental health care for people held in COVID-19 quarantine or isolation centres was reported to be scarce and not timely. One participant reported an unsuccessful effort to include mental health in the COVID-19 response at the level of regions and below, with low awareness and stigma in administrators and officials serving as an important barrier. Initially, mental health professionals were not an integral part of the COVID-19 health professional teams linked to quarantine, isolation and treatment centres. The national level COVID-19 task force included mental health professionals, but this aspect of the COVID-19 response was not operational at regional levels and below.

*Yes, especially individuals with unstable mental conditions in COVID-19 isolation were not getting mental health care. Health care providers fear people with mental illness. They avoid them or don’t want to engage to treat and support them because they suspect them as a source of risk for coronavirus infection. They also perceive them as a threat, causing harm because the health care providers have limited mental health literacy [Mental health expert; IDI12]*

Key informants reported that when people with known MHCs were admitted to COVID-19 treatment centres, health professionals withdrew from delivering the required care to the same standard as other patients without mental illness. This was related to their perceptions that people with MHCs would infect them or harm them.

At COVID-19 treatment centre, I saw one patient with bipolar disorder. I observed that HCWs did not approach her and listen to her complaints. They put her in a separate room. This might not be due to COVID-19 rather it was due to stigmatizing people with MHCs [Mental health expert; IDI02].

People with severe mental illness admitted to treatment centres, inadequate management of the person’s mental health state was reported to affect COVID-19 outcomes.

A woman with mental illness was admitted to COVID-19 treatment centre. Her baby was COVID-19 negative. Then it was a big mess to separate the baby from her. The mother was suspicious and believes others do harm her daughter. With the mess-up process of this, her baby was infected to COVID-19 because the baby was with her for a longer time before isolating the baby was done [Mental health expert; IDI13].

### Impact of COVID-19 on the physical health of people with MHCs

Intersecting vulnerabilities for coronavirus infection were reported for people with MHCs in the community. Due to the impoverishing effects of living with an MHC, many could not afford to sustainably buy protective equipment. Respondents also reported that people with MHCs were not able to access comprehensible information about COVID-19 that was tailored to their situation. There were only a few public health communication campaigns targeting people with MHCs, and this only happened about six months into the pandemic and was limited to Addis Ababa. Some audio-video messages, leaflets and posters designed for people with MHCs were disseminated at quarantine and isolation centres. People with MHCs were also not adequately protected from COVID-19 in institutional settings.

*Let me tell you the tragedy. Initially, almost more than half of the people with MHCs were showing COVID-19 symptoms. When we request COVID-19 testing, many found COVID-19 positive. Then we should have to admit them, but we were not allowed. Patients with acute disturbance and showing clear psychiatric symptoms were not allowed to be admitted and had no way to refer for COVID-19 treatment. People with MHCs were denied COVID-19 services. Leaders said they [people with MHCs] will disturb others if they get admitted with other people who are without MHCs. This was very sad. Finally, we were forced to admit a few of them with other psychiatric patients who were COVID-19 negative. So other psychiatric patients were systematically vulnerably to be infected. I personally was also infected* when working with them being without standard COVID-19 infection prevention protocol [Mental health expert; IDI14].

Mental health stigma was reported to have complicated the physical health care of people with MHC during the pandemic. Health care professionals feared infection with COVID-19 from people with MHCs. They perceived that people with MHCs did not properly wear face masks and might touch them or put them at risk for infection. As a consequence, health workers reportedly either neglected the physical health care needs of the person or they expected that people with MHCs should be restrained or held down by caregivers during the clinical consultation. There was a perception that long-standing problems with referring people with MHCs for physical health care were exacerbated during COVID-19. Social care institutions also suspended referring people with MHCs for physical health care during the early few months of the pandemic since health facilities were not fully functioning. Participants from primary health care and social care settings reported that they could only deliver essential or emergency health care services.

We [social care institution] suspended physical rehabilitation. Physiotherapy service has been completely discontinued which had been very important for our beneficiaries [Civic society representative; IDI05].

A mental health service user reported that since the health care facilities made operational changes to their services, people with MHCs were hardly accessing physical health care.

I know people with mental health conditions who came from the rural areas to get physical health care in Tikur Anbessa and St Paulo’s hospital but they went back without getting the service [Service user; IDI15].

In settings where physical and mental health care was integrated, like Dire Dawa Dilchora hospital, it was easier to sustainably deliver physical health care for people with MHCs during the pandemic. They only needed to make some modifications, including increased time between follow-up appointments, and were able to continue delivering both mental and physical health care. Where physical and mental health care was not integrated, long-standing mental health stigma among health care providers was perceived to complicate care and referral for physical health care. At mental health care facilities, COVID-19 services were reported to be inefficient. The settings were not safe and there were limited resources for COVID-19 infection prevention and control. Thus, the health facilities implemented problematic procedures, as perceived by clinicians.

*Our system of secluding people with MHC in our hospital looked problematic. Every patient suspected of COVID-19 would be in a room and did not allow being outside of the room. A patient would be allowed to go out from the secluded room only after testing for COVID-19. The intention is to avoid the risk of infecting other patients, but the process of taking a sample for COVID-19 testing has been taking some time; up to 2 to 4 days. For patients, staying in a secluded room had been very challenging and unacceptable [Mental health expert; IDI09].*

### Mental health services during COVID-19 outbreak

#### Availability and utilization of mental health care

Disruption to mental health care services because of the pandemic was reported at all levels of the health system. In many instances, mental health care had still not been restored to pre-pandemic levels at the time of reporting. As a result, the mental health treatment gap, which was already very high, had widened. In Addis Ababa, PHC facilities were not functioning to their full capacity during the early months of the pandemic due to many being dedicated as COVID-19 isolation centres. Redeployment of mental health professionals to the COVID-19 response resulted in a shortage of mental health professionals. For physical health care, professionals who were not working in the public health system were trained and deployed for the COVID-19 response, but this had little impact on mental health care since the numbers of mental health professionals in the private/NGO sector were small. During the pandemic, many hospitals back-referred people with MHCs to locally available PHC facilities. But since many primary health facilities did not have services for people with MHCs, this left a gap in the service. There were people with MHCs disorders who were attending follow-up care in PHC before COVID-19, but during COVID-19 mental health service utilization was reported very low. Participants reported that people with depression and anxiety disorders feared COVID-19 infection from health facilities and might prefer to stay at home.

Respondents reported that mental health services in general hospitals and mental health specialized hospitals were substantially disrupted by the pandemic. Many people with MHCs were discharged precipitously from in-patient care to decrease the risk of COVID-19 infection. Other in-patient mental health care facilities were designated as COVID-19 care facilities. Participants reported that the shift of the new Eka Kotebe hospital (which had 175 psychiatric beds pre-pandemic) to become a COVID-19 treatment facility was unexpected and unwelcome.

…and then we discharged all patients. Only a few of them with critical health conditions were referred to Amanuel hospital [the only dedicated psychiatric hospital]. We only have tried to resume the outpatient service at least on a partial scale. Previously, the hospital had about 175 beds for mental health services. After a month or so we managed to have 26 beds for patients with the psychiatric emergency, but only for males [Mental health expert; IDI13].

In addition to Eka Kotebe in Addis Ababa, other mental health facilities across Ethiopia were closed due to the pandemic, including the Catholic Church Missionaries of Charity and Sabian General Hospital in Dire Dawa and Karamara Hospital in the Somali region. Respondents perceived that this would result in many people with MHCs unable to access care and would increase the volume of people attending the remaining mental health care facilities. For example, when two hospitals were closed in a single city, the patient volume increased at another adjacent hospital. In other health facilities, mental health services were either partially reduced or suspended for several months during the pandemic.

In our hospital, patient volume was significantly reduced. We used to see about 400 to 500 patients per month before COVID-19, but during COVID-19, we only had to see about 200 patients per month on average [Mental health expert; IDI16].

In other mental health facilities, there was disruption of psychiatric emergency and substance use disorder rehabilitation services. In most settings, it was reported that rehabilitation services for people with substance use disorders and psychotherapy services had still not fully resumed at the time of reporting. Three addiction rehabilitation centres in Addis Ababa stayed closed for many months during COVID-19. Another rehabilitation centre remained open throughout the pandemic, but patient contact was reduced by half compared to usual.

In our hospital, the private wing was closed where many patients with emergency conditions were mainly getting services. The number of patients attending emergency services, in general, was also reduced. It creates a treatment gap [Mental health expert; IDI17].

Electroconvulsive therapy (ECT) was also discontinued from April 2020 – February 2021, meaning that some critically ill people could not access potentially life-saving care. Even after ECT was resumed, it was discontinued again temporarily in March 2021 after some people with MHCs were infected with coronavirus during the process of ECT due to lack of adequate infection control equipment and procedures. It was resumed after COVID-19 rapid test was made mandatory prior to receiving ECT.

Respondents reported that structural stigma towards mental health became evident during the pandemic, manifested in low political attention and commitment. They expressed their frustration that there had not been a more active resistance from mental health professionals and other mental health advocates.

We [Mental health care professionals] should have done a very organized voicing for people with mental health conditions and protect mental health resources. They [people with MHC] can’t label themselves saying “I am mentally ill”. There is a kind of double stigma. They will not be a voice for themselves. They need others to be a voice. We didn’t say anything when their resources were taken from them. We just clamp our hands and accept the decision. We didn’t say anything. We should have been strong advocates for the rights of people with mental health conditions. I found myself on the side of the oppressors or among those who introduced structural stigma for people with mental illness. So, next time, this should not be the case. We should not be like this [Mental health expert; IDI09].

Alongside reduced availability of mental health care, there was also reduced utilization of centralized mental health care facilities. This was reported to be largely driven by fear of infection, as well as the escalating transport costs. For example, at psychiatric nurse-led units in regional centres, while mental health care was available, monthly contact was reduced.

Overall patient flow was reported to be lower but new cases have increased at some health facilities. We [mental health care workers] used to report about 600 follow up patients per month, but it was reduced to 500 and later reached 400 [Mental health expert; IDI18].

**Fig. 1:**
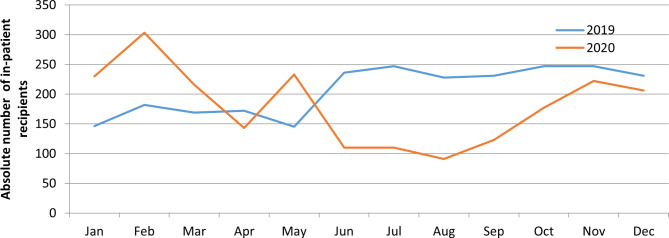
In-patient services utilization in Amanuel Mental Specialised Hospital, 2019 and 2020

At Amanual Mental Specialised Hospital in Addis Ababa, there was a statistically significant reduction in the number of admissions since March 2020 (Fig. 1), particularly during the state of emergency. The Health Management Information System (HMIS) in-patient data were reduced by about 60% for several months compared to 2019.

**Fig. 2:**
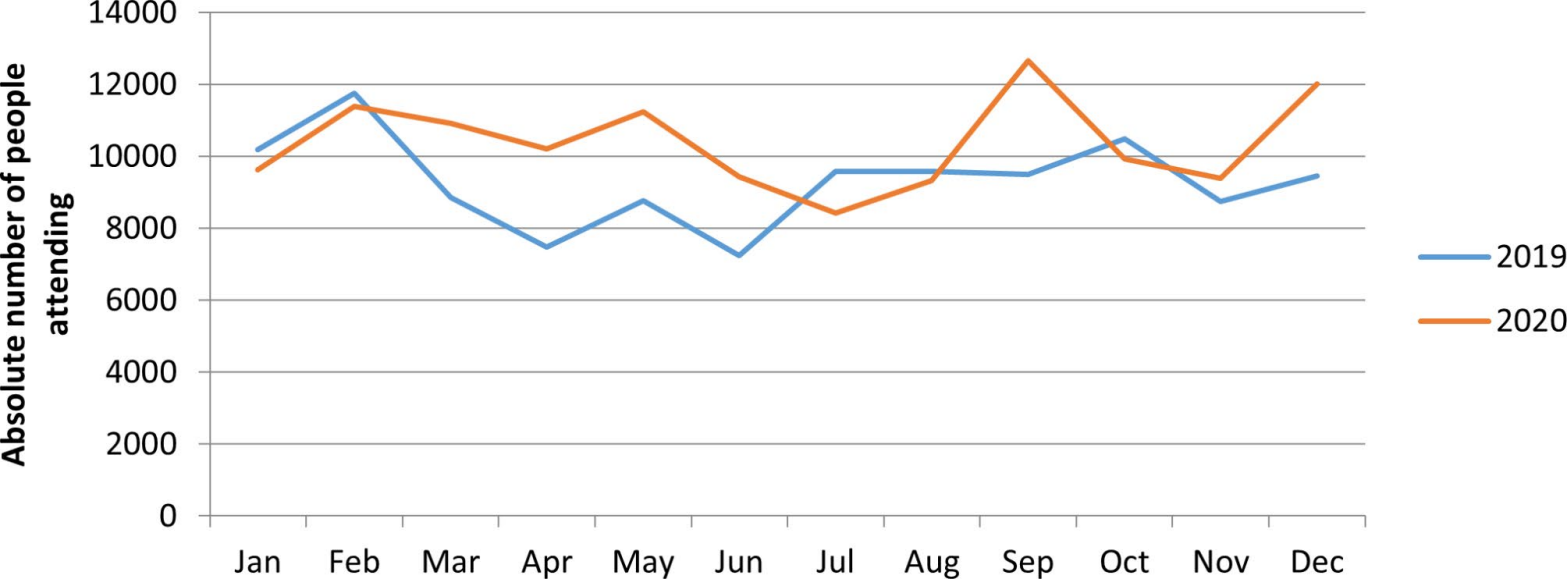
Outpatient service utilization in Amanuel Mental Specialised Hospital, 2019 and 2020

Given the closure of other mental health services, out-patient service utilization was expected to increase but actually remained relatively comparable for the years 2019 and 2020. See Fig. 2.

Over a period of a few months from July to August in 2020, there was a decline in in-patient service utilization, with the number of patients falling to 100 compared to the same period in 2019. In contrast, from February to June in 2020, out-patient service utilization increased compared to 2019.

**Table 2:** Results of the segmented poisson regression comparing mental health service utilization before and after the pandemic

	In-patient service utilization		Out-patient service utilization	
	AIRR*	95%CI	AIRR	95%CI
Time since the 1st month of 2019 to the last month of 2020	1.03	1.01, 1.05	1.01	0.99, 1.02
Time when there is no COVID-19 and where there is the incidence (dichotomous measure)	0.74	0.50, 1.09	1.19	1.01, 1.40
Incidence of state of emergency (dichotomous measure)	0.98	0.64, 1.48	0.86	0.77, 0.97
12 months period since COVID-19 incidence	0.95	0.89, 1.01	0.99	0.96, 1.01

*AIRR: Adjusted Incidence Risk Ratio

The segmented poisson regression results (Table 2) show an increasing trend in mental health service utilization both prior and after the COVID-19 pandemic, but that inpatient mental service utilization was significantly decreased by the introduction of the nation-wide state of emergency (AIRR = 0.86; 95% confidence interval 0.77, 0.97).

### Quality of mental health care during COVID-19 outbreak

As well as reduced availability of mental health care, respondents also reported concerns about the quality of mental health care. One of the indicators for poor mental health quality was the long periods between follow-up appointments, up to six months in some cases. This meant that people with MHCs were not able to be actively engaged in their care through discussions with mental health professionals. If they experienced medication side effects, they had no opportunity to consult their mental health care provider.

We [clinicians] were forced to appoint patients after six months. This had a negative impact on the quality of mental health care. It is impossible to know whether the medications work or not and whether medications induce side effects or not [Mental health expert; IDI17].

The absence of family visits to people receiving in-patient care and the lack of recreational activities compromised the quality of mental health care.

Clinicians also reported challenges with conducting psychiatric assessments and consultations. Face masks and physical distancing were considered barriers to assessment and therapeutic communication. In a few private mental health facilities, psychiatrists interviewed people with MHCs using mirrors so that they could attend to non-verbal communication. But such initiatives were not reported in public mental health facilities. A psychiatrist reported his experience as:

Patients had not been getting appropriate service during the early months of the pandemic. Although many patients didn’t come, those who visited us were also not properly assessed and consulted. Both of us, the patient and the clinician, were talking wearing face mask. In a psychiatric interview, it is difficult to know the external emotional changes over the face of the patients. It is difficult to understand non-verbal communication since face mask is a barrier. In addition, due to fear of the infection, we interview patients for a limited brief period of time. Due to the risk of infection, we also rarely engage caregivers during the clinical interview. Thus, we are only able to get a limited amount of information about their illness [Mental health expert; IDI09].

## Discussion

The mental health response to the coronavirus pandemic in Ethiopia was hampered by pre-pandemic scarcity and inaccessibility of mental health care services alongside systemic mental health stigma and discrimination that has reinforced low prioritization of mental health. Our study shows that, despite the increasing mental health needs in Ethiopia during the pandemic, the availability of mental health care diminished, resulting in a widening of the mental health treatment gap. As well as mental health effects in the general population, the pandemic resulted in poorer mental and physical health, economic hardship, and human rights abuses among people with existing mental health conditions and their family members.Despite a large number of reports from Ethiopia on the impacts of the pandemic on the general population and health workers, we were unable to find any studies examining the impacts on mental health services and people with existing mental health conditions.

Upon reviewing existing studies on mental health and COVID-19 in Ethiopia, a systematic review estimated a prevalence of 42.5% for psychological distress associated with COVID-19 [39]. Among healthcare professionals, anxiety symptoms were found to be 46%, while overall stress levels were about 51% [40]. This percentage is higher compared to the reported prevalence of anxiety symptoms among individuals in the informal sector in Ethiopia, which is about 32% [41]. The reviews also highlighted that high levels of mental distress were associated with factors such as age, sex, marital status, lack of social support, substance use, other comorbid medical conditions, working department, history of contact with confirmed COVID-19 cases, and lack of protective equipment. However, a previous study focusing on the consequences of COVID-19 among people with mental health conditions reported increased vulnerability to infection, economic problems, discrimination, and difficulties accessing care among this population [37].

Disruption of mental health services were reported in Ethiopia which was also seen early in the pandemic globally [42]. There were reports of a number of different innovations in service delivery to try to ensure continuity of care both in high-income countries and LMICs [43], particularly relying on digital technologies. However, in Ethiopia, such innovations were rarely reported and were limited to the private sector. This reflects the relatively low accessibility and unreliability of internet-based platforms in the country [44], alongside low literacy. This ‘digital divide’ has been highlighted by the World Health Organization, whereby the people with greatest need for mental health care may be differentially excluded from access to digital technologies [42].

Before the pandemic hit Ethiopia, the consequences of low population awareness of mental health, stigma and low access to care were already profound [45, 46]. Combined with the pandemic, it appears that more households of people with MHCs have been pushed into poverty, with worsening of stigma and human rights abuses, and increased risk of abandonment and homelessness of people with MHCs [47, 48]. Our findings of differential impacts of the pandemic upon people with existing severe mental health conditions is echoed by other studies. In China, adverse effects of the COVID-19 pandemic on people with severe mental health conditions were reported, including deterioration in symptoms or relapse, increased risk of COVID-19 infection, and partiality in the provision of physical healthcare [49]. Social restrictions linked to the pandemic were found to exacerbate mental health-related stigma and social isolation in people with severe mental health conditions, compounded by problems with access to tailored information about COVID-19 [1].

The historic separation of mental health and physical health care in Ethiopia meant that the country was slow to ensure that the mental health and psychosocial needs of people in COVID-19 isolation and treatment centers were met, while also neglecting to protect people receiving mental health care from COVID-19. Structural discrimination is likely to have played a part in the difficulties with access to physical health care experienced by people with existing mental health conditions [50]. Previous studies in Ethiopia have reported on the barriers to quality mental health care due to out-of-pocket payments for treatment, interrupted supplies of psychotropic medications, inaccessibility of mental health care, poor mental health management information systems, poor engagement of service users in service development and improvement, and the lack of mental health legislation [22, 51, 52].

COVID-19 prevention strategies are in stark opposition to what is recommended to facilitate recovery in people with mental health conditions [53]. COVID-19 prevention strategies such as maintaining social distance or lockdowns, also affected both the engagement of caregivers in mental health care and exacerbate psychiatric symptoms among people with pre-existing mental health conditions. From the health care providers as well, mental health assessment and therapy were compromised by the COVID-19 prevention strategies in clinical settings, leading to a deterioration in mental health especially among individuals with severe mental illness.

Our findings have exposed the neglect of mental health care and people with MHCs in Ethiopia during the pandemic. It is essential to mobilize political commitment and population support to implement the National Mental Health Strategy in full [17]. The integration of mental health into primary and general health care services would make an important contribution to increasing health system resilience in the face of future emergencies, whether they are public health emergencies, conflict-related or due to other humanitarian crises. If people with mental health conditions can access care in local health centres, this will greatly increase access to care even when movement is restricted, as in a pandemic. Integrating physical and mental health care also makes sense in response to the high co-morbidity [54], as well as providing an opportunity to address the high levels of unmet physical health needs in people with severe mental health conditions [13]. The evidence for the effectiveness, safety and acceptability of integrating mental health into primary care in Ethiopia is strong [25]. Furthermore, blueprints for how to scale up this model of care in Ethiopia and other low-income countries already exist [55]. In addition to local evidence, international initiatives such as the WHO Comprehensive Mental Health Action Plan emphasize the need for integrating mental health services into community platforms and primary healthcare settings. As part of a global target, 80% of countries are expected to double the number of community-based mental health facilities by 2030 [56]. As a member state, Ethiopia has committed to these goals, and there should be a strong willingness and commitment to implement the evidence and initiatives. A limitation of this study was the small number of mental health service users who participated. This was because of our focus on national level impacts, so that we included a representative of mental health service users. This work complements our previous work exploring impacts of the pandemic on people with lived experience in a more local setting [37].

## Conclusions

The COVID-19 pandemic has exacted substantial negative effects on both the lives of people with mental health conditions and mental health care in Ethiopia. The response to any public health emergency in Ethiopia should make mental health an integral part of the planning, organization and multisectoral actions. Furthermore, people with mental health conditions should be prioritized as a vulnerable group needing additional economic and social support and tailored health promotion and disease prevention services. Integrating mental health into primary and general health care is a key requirement to increase the resilience of mental health care systems to future emergencies.

### Electronic supplementary material

Below is the link to the electronic supplementary material.

**Supplementary Material 1**: MASC narrative framework

## Data Availability

All data generated or analyzed during this study are included in this article. Further inquiries can be directed to the corresponding author.

## Abbreviations

COVID-19: Coronavirus disease
ECT: Electroconvulsive Therapy
EHRC: Ethiopian Human Right Commission
EPSA: Ethiopian Pharmaceuticals Supply Agency
HCW: Health care worker
HEW: Health Extension Worker
HMIS: Health Management Information System
IDI: In-depth interview
MHPSS: Mental Health Psychosocial Support
MHSUA: Mental Health Service Users Association
MoH: Ministry of Health
PHC: Primary Health Care
MHC: Mental Health Conditions
PPE: Personal Protective Equipment
TASH: Tikur Anbessa Specialized Hospital
